# Role of Biomarkers as Prognostic Factors in Acute Peripheral Facial Palsy

**DOI:** 10.3390/ijms23010307

**Published:** 2021-12-28

**Authors:** Tae Hoon Kim, Seung Geun Yeo, Jae Yong Byun

**Affiliations:** Department of Otorhinolaryngology—Head and Neck Surgery, School of Medicine, Kyung Hee University, Seoul 05278, Korea; niceyozm@gmail.com (T.H.K.); yeo2park@gmail.com (S.G.Y.)

**Keywords:** acute peripheral facial palsy, Bell’s palsy, Ramsay Hunt syndrome, biomarkers, prognosis

## Abstract

Acute peripheral facial palsy (APFP), including Bell’s palsy and Ramsay Hunt syndrome, is a disease that affects daily life through facial motor dysfunction, causing psychological problems. Various tests to evaluate prognosis have been studied; however, there are no validated predictive biomarkers to guide clinical decision making. Therefore, specific biomarkers that respond to treatment are required to understand prognostic outcomes. In this review, we discuss existing literature regarding the role of APFP biomarkers in prognosis and recovery. We searched the PubMed, EMBASE, and Cochrane Library databases for relevant papers. Our screening identified relevant studies and biomarkers correlating with the identification of predictive biomarkers. Only studies published between January 2000 and October 2021 were included. Our search identified 5835 abstracts, of which 35 were selected. All biomarker samples were obtained from blood and were used in the evaluation of disease severity and prognosis associated with recovery. These biomarkers have been effective prognostic or predictive factors under various conditions. Finally, we classified them into five categories. There is no consensus in the literature on the correlation between outcomes and prognostic factors for APFP. Furthermore, the correlation between hematologic laboratory values and APFP prognosis remains unclear. However, it is important to identify new methods for improving the accuracy of facial paralysis prognosis prediction. Therefore, we systematically evaluated prognostic and potentially predictive APFP biomarkers. Unfortunately, a predictive biomarker validating APFP prognosis remains unknown. More prospective studies are required to reveal and identify promising biomarkers providing accurate prognosis.

## 1. Introduction

Acute peripheral facial palsy (APFP), the seventh cranial nerve palsy, is the sudden weakening of facial muscle movement on one side of the face. It appears as idiopathic facial paralysis in the majority of cases, of which Bell’s palsy (BP) is the most common form, accounting for 60–75% of all incidences [1]. The estimated lifetime risk of developing Bell’s palsy for individuals is 1 in 60, and the annual incidence rate is approximately 11–40 per 100,000 people [2]. Ramsay Hunt syndrome (RHS) is the second most common cause of APFP. It is characterized by paralysis of the facial nerve, otalgia, vesicles, and rashes occurring in the affected ear.

The pathogenesis of APFP is unclear; however, it is widely recognized as a result of reactivation of herpes simplex virus type 1 and varicella-zoster virus [3]. In addition to viruses, there are other potential causes, such as tumors, trauma, anatomical abnormalities, inflammation, ischemia, and acute cold exposure [4,5]. For APFP treatment, there is consensus that early use of prednisolone is effective; however, the use of antiviral drugs remains controversial. Moreover, combined steroid and antiviral therapy is more effective in patients with severe BP [6]. The response of patients to treatment and prognosis varies. Despite the multifactorial factors of the disease, majority of patients resolve between 3 and 6 months, though certain patients never recover completely [7].

The initial degree of facial weakness is important for providing prognostic information for facial recovery. The House-Brackmann grading system (HBS) and Sunnybrook scales are commonly used to quantify facial weakness severity, and recently, the eFACE system, which is measured with a smartphone [8]. A grading system that evaluates facial movements is subjective and inconsistent depending on the clinician [9].

To date, accurate prediction of APFP prognosis is challenging for physicians. Electrophysiological tests such as electroneurography (ENoG), stapedial muscle reflex, blink reflex, nerve excitability test, and electromyography (EMG) have been useful. ENoG has a significant correlation with prognosis and is used when considering facial nerve decompression surgery [10]. EMG can assist in determining the presence of nerve damage and determine its severity. However, these tests are occasionally clinically unsuitable because they must take place over a specific time period [11]. Therefore, another indicator that can predict prognosis is required.

Considering the above-mentioned etiologic factors of APFP development, a laboratory test that evaluates prognosis is considered desirable. Numerous studies have investigated hematologic markers based on inflammatory mediators, vascular failure, oxidative stress, and immunological responses [12,13,14,15].

**Figure 1:**
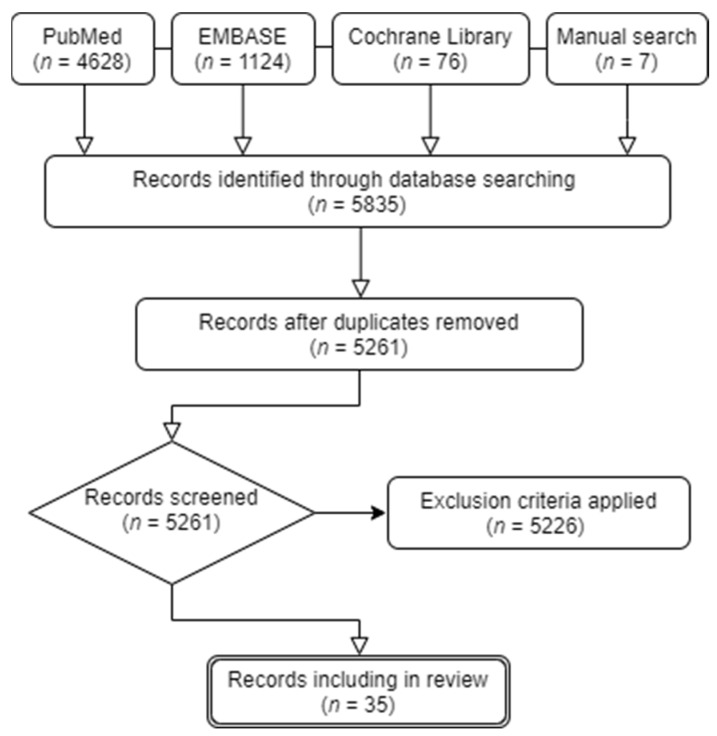
Diagram showing selection of studies for review.

**Figure 2:**
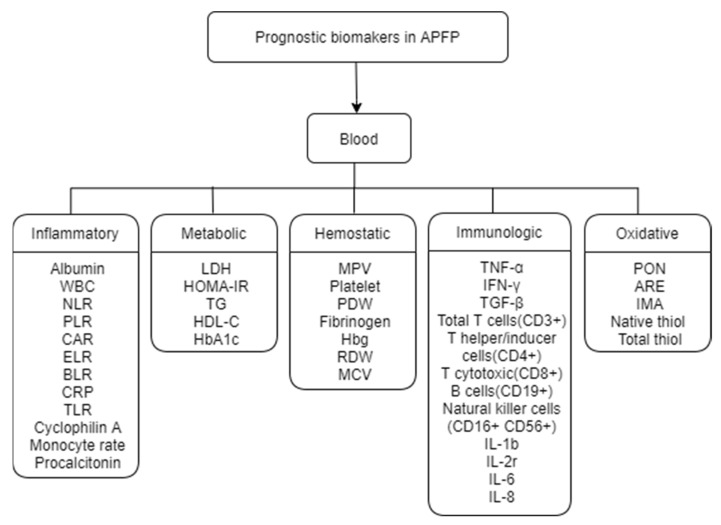
Diagram illustrating the review biomarkers.

Potentially eligible studies were identified in a search of the US National Library of Medicine electronic database (PubMed), EMBASE, and Cochrane Library, using a combination of the following terms: “Facial palsy”, “Bell’s palsy”, “Ramsay-hunt syndrome”, “biomarker”, “prognosis”, and “factor.” Additionally, studies were included in the studies using a manual search method. Only studies published after 2000 were included in the analysis. The initial search was performed in April 2021 and updated in October 2021. Studies were selected for review based on the following criteria: (1) studies investigating the association between APFP and biomarkers; (2) in English; and (3) studies reporting outcomes based on prognostic and/or predictive biomarkers. Studies were excluded if they met the following criteria: (1) not in English; (2) duplicate publications; (3) meta-analyses and systemic reviews; (4) no human study population; (5) unclear clinical implications; and (6) not related to the review topic. Finally, we included possibly relevant studies that were screened to confirm their eligibility. A literature search identified 5835 studies. After initial title screening and manual reduplication, 574 studies were excluded (not relevant to the topic), and 5261 records remained for abstract review. We performed a full abstract review evaluation of the remaining studies by applying exclusion criteria. Thirty-five studies that investigated prognostic and predictive biomarkers for APFP were finally included in this study (Figure 1). In all studies, the potential of blood biomarkers for APFP was evaluated. Since biomarkers were evaluated using various techniques and there is no standard categorization for AFPF biomarkers, prognostic biomarkers were subdivided as follows: (1) inflammatory, (2) metabolic, (3) hemostatic, (4) immunological, and (5) oxidative (Figure 2). Information regarding the studied biomarkers, including study design, sample size, age, and results/conclusions, are listed in table format (Table 1, Table 2, Table 3, Table 4 and Table 5).

To our knowledge, very few potential biomarkers have actually proceeded towards the path of validation, although many have been proposed. This review is the first to list the published literature on biomarkers used to predict APFP prognosis. Our focus was on biomarkers related to the following question: What are the currently available prognostic biomarkers that aid in predicting the clinical outcomes of APFP? In this way, we aimed to identify and pursue the most promising prognostic biomarkers for further evaluation and validation studies.

## 2. Prognostic Biomarkers in APFP

### 2.1. Inflammatory

Inflammation theory suggests that when the facial nerve becomes inflamed, the nerve sheet thickens and the nerve jam swells in the fallopian canal, particularly in the labyrinth segment [47]. The majority of studies have focused on the association between inflammatory parameters and biomarkers. Immune system cells, including lymphocytes, neutrophils, and monocytes, and cell-mediated inflammatory responses are recognized as important for tumorigenesis and carcinogenesis [33].

The neutrophil-to-lymphocyte ratio (NLR) is a commonly used inflammatory marker that appears in all 23 inflammatory studies we reviewed. It can be used to diagnose and follow up on various inflammatory diseases, and has been found to provide useful information on the prognosis of these diseases [13]. Furthermore, NLR has been used to evaluate the risk of cardiovascular disease, and the prognosis of patients with cardiovascular disease and various cancers [48,49]. Other studies have investigated the association between BP and various hematological parameters. C-reactive protein (CRP) is a positive acute-phase reactant used for the diagnosis and evaluation of therapeutic efficacy in patients with infection/inflammation [50]. In general, albumin is known to decrease in acute inflammation, however, in chronic processes of inflammation, it is also seen in malnourished patients [51]. CRP and albumin have prognostic value for inflammation in the short- or long-term [52]. Procalcitonin, a precursor of the hormone calcitonin, is produced by C cells in the thyroid gland and neuroendocrine cells in the lungs or intestine. It is currently the most frequently used pro-inflammatory biomarker in clinical practice [53]. A new inflammatory index, the systemic immune-inflammation index (SII), has been shown to be a prognostic marker for malignant tumors and inflammatory diseases, where SII = platelets × neutrophils/lymphocytes [54]. Cyclophilin A (CyPA) is secreted in response to inflammatory stimuli, such as infection, hypoxia, and oxidative stress [55].

It is widely known that NLR levels are highly correlated with BP prognosis [32,38]; certain studies have reported a higher NLR in BP patients experiencing poor outcomes [38], a positive correlation between NLR and disease severity [14,33,36,56], and longer recovery times in patients with a higher NLR [25]. Additionally, facial nerve contrast enhancement was confirmed by MRI in patients with a high NLR [36]. A study on RHS patients revealed that a high NLR was associated with poor outcome of facial palsy, and showed a lower rate of complete recovery than that of the normal NLR group [26]. It was observed in a Japanese study that the NLR of all subjects in the RHS group was significantly higher than that in the BP group, with a lower recovery rate. The severity of inflammation caused by viral infection has been shown to correlate with the prognosis of facial paralysis. In a previous study examining the correlation between the C-reactive protein to albumin ratio (CAR) and certain disorders, such as chronic sinusitis, nasal polyposis, various cancers, and inflammatory diseases, CAR was reported as a valuable prognostic parameter [57]. Similarly, one study concluded that CAR, rather than other values, was an indicator of poor BP prognosis [20]. Procalcitonin levels were also significantly correlated with HBS and poor recovery, which may provide important information on facial nerve inflammation [31]. In the analysis using SII, the SII value was better indicative of BP than the NLR value. The SII has a higher specific and positive predictive value than NLR [21]. In addition, unlike EMG, serum CyPA concentration can be measured in the early phases of the disease and is an early marker that could have important implications. Although the recovery period CyPA levels cannot be predicted through observation, our results show that low CyPA levels indicate a shorter average recovery time than high CyPA levels [23].

Contrary to previous studies reporting that RHS patients with a high NLR experienced poor outcomes, NLR may only have a limited prognostic role in RHS patients [26]. For BP, NLR was not associated with its severity or prognosis. A group in Turkey reported that NLR and platelet-to-lymphocyte ratio (PLR) may be related to BP; however, no significant relationship was detected in BP prognosis [35]. Another study reported that only the NLR ratio value of the RHS group was higher than that of the control group; however, there was no statistically significant association with HBS [30]. In a Japanese study, the NLR was higher in the RHS group than in the BP group, suggesting that the severity of inflammation caused by viral infection is correlated with prognosis [32]. Inflammation plays an important role in the pathogenesis of RHS. The initial RHS severity and response to corticosteroids may determine the final treatment outcome. However, inflammation markers were not predictive of all BP outcomes. BP may be etiologically heterogeneous [18].

Prognosis and prognosis-related factors have been applied in various parameters in adults, however, studies on children are limited, and reliable recovery evaluation methods are lacking. In facial paralysis patients, ENoG and EMG are useful tools for predicting prognosis; however, in children, this prediction is difficult because of limited cooperation. There are reports that there is no difference between the pediatric population and adults in terms of these pathophysiological causes [58]. Children have a high rate of complete recovery and are generally characterized by acute onset and non-rapid self-limiting features [59]. Consistent with the evidence in the literature, significantly higher NLRs were identified in pediatric patients than in controls in a recent study. Furthermore, the authors postulated that the NLR was significantly higher in the non-recovery group than in the recovery group, which may indicate poor prognosis in pediatric BP patients [17]. According to the results obtained from the receiver operating characteristics analysis, it was determined that CAR (0.879) had higher area under the curve values than those of NLR (0.794). Therefore, CAR is predicted to be a more valuable prognostic marker than NLR in pediatric facial paralysis. In was seen in another pediatric study that the higher the grade of facial paralysis, the higher the NLR ratio. However, there was no significant relationship between mean platelet volume (MPV) and thrombocyte-to-lymphocyte ratio (TLR) values and severe facial paralysis [22]. There was a positive correlation between NLR and HBS. PLR values were also found to be positively correlated with HBS. However, a positive correlation could not be demonstrated in MPV and red cell distribution width (RDW) values [24]. One study reported that NLR cannot be an early recovery value because variability (such as patient age, heritability, and weather) occurs due to genetic and environmental factors [60].

Serum albumin is the most abundant protein in the plasma, with various essential biological functions, and is a well-known nutritional and inflammatory marker [61,62]. Under inflammatory conditions, the vascular endothelial permeability increases. When various components in blood vessels, including albumin, permeate the tissue space, the concentration of albumin in the plasma decreases [63]. Therefore, low albumin levels may indicate persistent inflammation. The clinical significance of serum albumin in prediction has been reported for the prognosis of certain diseases, including ulcerative colitis, cirrhosis, and cardiovascular disease [64,65,66]. It was clear that serum albumin levels were positively correlated with the prognosis of Bell’s palsy patients. The number of days hospitalized was negatively correlated with the recovery of Bell’s palsy [16].

### 2.2. Metabolic

Schwann cells and myelin sheaths are more likely to be affected in diabetic patients than in non-diabetic patients. Chronic hyperinsulinemia in diabetic patients may cause a decrease in endoneural oxygen, blood flow, and extraneural arteriovenous shunt and compensatory response to intimal ischemia/hypoxia, all of which can contribute to chronic nerve ischemia [41]. The prognosis of individual BP patients is generally good, however, diabetes is a potential etiologic and poor prognostic factor for BP [67]. There was no difference in HBS between the diabetic and nondiabetic groups at the onset of treatment, and at 1 month after onset. However, it was confirmed that the diabetic group had poor facial movements at 3 and 6 months. The recovery rate after six months was also lower in the diabetic group [42]. Adour et al. recommend a blood glucose elevation test for patients with recurrent facial paralysis given the frequency of diabetes mellitus (DM) in BP patients [68]. Conversely, multivariate analysis suggested that DM-related decreased blood circulation may negatively affect the prognosis of facial paralysis patients, and a recent study on diabetes and facial paralysis recovery time reported that diabetes had a partial effect on BP prognosis [69]. There was no significant difference in the complete BP recovery rate between the diabetic and non-diabetic groups [70]. Similarly, patients with abnormal HbA1c values had more HBS V/VI, however, follow-up results after 6 months were not correlated with prognosis [41].

Prediabetes, defined as impaired fasting glycemia and impaired glucose tolerance associated with insulin resistance (IR), increases the risk of developing diabetes [71]. The pathogenesis of diabetes or prediabetes neuropathy is understood as an IR-related mechanism for neuronal injury associated with the insensitivity of neurotropic properties to insulin [72]. In addition to identifying more accurate prognostic factors, studies have used the Homeostatic Model Assessment for IR (HOMA-IR) to estimate IR in non-diabetic facial palsy patients [39]. HOMA-IR used for evaluation of insulin sensitivity was calculated using the following formula: basal plasma glucose (mg/dL) × basal plasma insulin (UI/mL)/405. The results showed that patients with higher HOMA-IR values had a higher HBS, suggesting slower recovery than those with low HOMA-IR values.

Lactate dehydrogenase (LDH) is found in the cytoplasm of all human tissues. During inflammation-mediated cell damage, lactic acid is converted to pyruvic acid and released extracellularly. LDH has previously been used as an infectious disease biomarker in inflammatory diseases [73,74]. Serum LDH concentrations were significantly higher in the BP group than in the control group. This finding could be considered an indicator of inflammatory disease during BP development [19].

Metabolic syndrome (MetS) is a chronic metabolic disease characterized by the manifestation of various symptoms, such as impaired glucose tolerance, hypertension, hyperlipidemia, and obesity. MetS is associated with an increased risk of DM, myocardial infarction, stroke, and cardiovascular mortality, and is directly or indirectly involved in the development of various other diseases [75]. MetS induces various types of neuropathy through nitrous oxide inhibition, vascular degeneration, oxidative injury, and the pathological mechanisms involved. The complete recovery rate determined by the final HBS was lower in the MetS group than in the non-MetS group. The recovery rate in the MetS group was significantly lower in patients with DM, obesity, or high triglyceride (TG) levels [40]. TG and high-density lipoprotein cholesterol (HDL-C) are associated with atherosclerosis. Microcirculation disorders occur due to an increase in fat and cholesterol accumulation in the lining of the blood vessel wall. This, in turn, leads to endothelial dysfunction and vascular inflammation, leading to plaque formation, vascular remodeling, and vascular luminal obstruction, eventually resulting in microvascular ischemia and facial paralysis. [76].

### 2.3. Hemostatic

The ischemia theory suggests an impaired blood circulation. Vasa nervorum, caused by blood clots or vasospasm, causes nerve damage [77]. MPV is an indicator of platelet function and indicates the rate at which platelets are produced. High MPV values are associated with ischemic vascular conditions, such as atherosclerosis and thromboembolism [78]. The MPV value shows the platelet size, while the PDW value is used to evaluate variations in platelet size [79]. PLR has been proposed as a marker for ischemic disorders. Patients with increased PLR values have been found to be at high risk for several peripheral vascular and coronary artery diseases [80].

In a comparative study between the recovery and non-recovery groups, PDW values were significantly higher and platelet counts were lower in the recovered group than in the unrecovered group [28]. PLR was higher in BP patients than in healthy controls. However, no correlation was found between PLR and BP grades [35]. In another study, MPV was found to be higher in BP patients. Resultingly, the authors found that higher MPV and PDW values were associated with HBS. However, they reported that there was no correlation between MPV and PDW values and the prognosis of facial paralysis [14]. Meanwhile, one study did not find higher MPV and PLR values in BP patients than in controls [19]. In addition, previous studies have reported no correlation between MPV and HBS [30,36]. MPV values also vary greatly depending on sex, age, and ethnicity. Therefore, it is argued that the MPV value is inconducive for disease diagnosis and prognosis prediction [81]. Plasma fibrinogen, an important coagulation factor, is synthesized in the liver and converted to fibrin by activated thrombin. In addition, it is a modest acute phase response protein that accumulates in response to many types of tissue damage and inflammation [82]. In a Chinese study, it was reported that plasma fibrinogen levels increased with an increase in HBS. In particular, the levels of HBS-VI were the highest [43]. It was suggested that increasing fibrinogen content increases blood viscosity, which aggravates the impaired facial nerve microcirculation.

The RDW and mean corpuscular volume (MCV) are parameters that describe the diameter and variation in red blood cell volume. RDW increases in anemia, hemolytic, microvascular thrombosis, and inflammatory diseases [83,84]. Inflammatory changes and erythropoiesis can increase RDW because immature red blood cells are released into the peripheral circulation [83]. MCV is calculated by multiplying the percent hematocrit by ten divided by the erythrocyte count, which is useful in determining the etiology of anemia [85].

A recent study showed RDW to be significantly higher in non-recovered patients than in recovered patients. The authors suggested that RDW could be a useful prognostic factor in BP patients. In multiple logistic regression analysis, only RDW had a significant effect on the likelihood of recovery from BP. Multivariate analysis results further indicated that only RDW was an independent prognostic factor for BP recovery [29]. Even when the MCV values were compared, there was a significant difference between the recovered and unrecovered groups [32]. The Yanagihara grading system was used to evaluate facial movements; this grading system is most commonly used in Japan, and is similar to the Sunnybrook scales and HBS [86]. In contrast, in other studies, RDW values were not significantly higher in BP patients [30]. Similarly, in pediatric patients, the RDW value was not correlated with prognosis [24]. A pediatric study suggested that RDW was unaffected by age, sex, and asymptomatic nutritional anemia, and therefore, was not associated with early recovery values [27].

Here, we report on the development and testing of a new scoring system, and the resulting simple scores (PPP-H score for RHS and PPP-B score for BP) to predict the prognosis of facial palsy patients. It consists of pre-treatment hematological biomarkers and patient profiles. We selected age, sex, and NLR to construct the PPP-B score, and age, monocyte rate, MCV, and platelet count to construct the PPP-H score. Resultingly, the PPP-B score included age (≥65 years), sex (male), and NLR ratio (≥2.9). The PPP-H score included age (≥50 years), monocyte rate (≥6.0%), mean corpuscular volume (≥95 fl), and platelet count (≤200,000/L). The patient recovery rate significantly decreased with increasing PPP scores (both PPP-B and PPP-H) in a stepwise manner [7].

### 2.4. Immunologic

The primary factors responsible for the elimination of viral infections is antibody-dependent cytotoxicity resulting from cellular and humoral immune responses. Interleukin (IL) and tumor necrosis factor alpha (TNF-α) cytokines are secreted by specific cells (including, monocytes, endothelial cells, epithelial cells, T cells, and natural killer cells). These cytokines are involved in immune responses, inflammation, acute phase reactions, and fever. The proinflammatory cytokines IL-1β, TNF-α, and IL-6 can be secreted by glial cells. Transient expression of these molecules triggers secondary events conducive to the repair and regeneration of the facial nerve [87]. In cases where the patient has a low CD19+ cell count, viral clearance is completely or partially reduced as a result of insufficient antiviral immune antibodies. Therefore, a decreased CD19 level such as in the case of B lymphopenia may be the cause of facial paralysis [88]. Moreover, depression of CD4 subsets leads to prolonged viral clearance and an autoimmune response to targeted tissue [89]. A study on adult patients revealed that there were changes in lymphocyte subsets in the peripheral blood during the acute phase of BP [90]. Decreased percentages of total T cells (CD3) and T helper/inducer cells (CD4) have been documented in the acute phase of BP [91]. Serum concentrations of inflammatory markers, such as TNF-α, IL-6, and IL-8, were increased in BP patients [45,92]. Additionally, IL-6, IL-8, and TNF-α levels were significantly higher in patients with Bell’s palsy than in controls, and IL-1β and IL-2r levels were similar in both groups. However, levels of the cytokines IL-6, IL-8, TNF-α, IL-1β, and IL-2r did not correlate with recovery [45]. In a pediatric patient study, lower rates of CD19 and CD4 levels were found in the child BP group than in the age-matched healthy control group. However, after 3 months, there was no significant difference in peripheral blood lymphocyte subsets between the satisfactory recovery group and the unsatisfactory group [15]. Overall, cytokines cannot function or be stored in cells. Therefore, in response to facial paralysis, levels may change owing to cytokine production. This may be helpful in understanding the pathogenic factors and underlying pathology mediating BP [45].

### 2.5. Oxidative

Oxidative stress begins as a result of impaired antioxidant defense and the balance of reactive oxygen species (ROS), and has been identified as a key step in the pathophysiology of vascular diseases. A few small intrinsic blood vessels exist in the labyrinthine segment of the facial nerve compared to the mastoid and tympanic segments. Thus, the labyrinthine segment may be more susceptible to oxidative stress. Thiols, which consist of a sulfur atom and a hydrogen atom bound to a carbon atom, are functional sulfhydryl groups [93]. The thiol group of proteins, such as albumin, is oxidized by molecular oxygen and reversibly converted to a disulfide bond. These disulfide bonds can be reduced to thiol groups under conditions of reduced oxidative stress [94]. Alterations in the thiol/disulfide balance contribute to antioxidant protection, detoxification, enzyme activity, and regulation of cell signaling mechanisms [95]. The mean native thiol and total thiol levels and native thiol/total thiol ratios were lower in the study group than in the control group. However, there was no correlation between HBS and thiol profiles [12]. Paraoxonase 1 (PON1) is an enzyme with a glycoprotein structure that has both PON and arylesterase (ARE) activities [96]. PON1 is an antioxidant enzyme linked to HDL and plays a role in protecting low-density lipoprotein from free radicals [97]. Systemic markers of oxidative stress include ischemia-modified albumin (IMA). When tissue ischemia occurs, a newly formed albumin called IMA is produced [98]. In BP patients, PON and ARE activities decreased, and IMA increased due to excess ROS [46]. The exact mechanism of this numerical change is unknown; however, it will provide information on the relationship between oxidative stress and BP.

## 3. Conclusions

Biomarkers play an important role in APFP prognosis, but the developmental path toward a clinically valid biomarker is always long and challenging. In this review, we divided biomarkers into five categories and described those that are helpful in APFP prognosis; however, we could not identify biomarkers with clinically distinct promising validity. Notably, these biomarkers have demonstrated different clinical values. All the biomarkers discussed in this review have the advantage that they can be obtained from blood and can be easily collected from routine tests. However, studies on biomarkers that can be obtained from the central nervous system, such as cerebrospinal fluid, should also be reviewed. In addition to biomarker studies focusing on inflammatory parameters, more extensive prospective studies such as those on endocrine and neurological parameters are needed. In this way, we will be able to acquire information that will facilitate better clinical decision making and apply it to the treatment of patients.

## Figures and Tables

**Table 1:** Summary of prognostic biomarkers for acute peripheral facial palsy (Inflammatory parameter).

Author (Year)	Study Design	Sample Size	Age (years)	Biomarkers	Results/Conclusions
Shang et al. (2021) [16]	Retrospective	311 patients	39.7 ± 11.8 (Mean)	Albumin	The serum albumin level of patients with BP in the unrecovered group was significantly lower than that of the recovered group
Cayir et al. (2021) [17]	Retrospective	59 patients, 65 controls	10.69 ± 5.76, 10.87 ± 3.22 (Mean)	WBC, NLR, PLR, CAR	High NLR and CAR values in pediatric BP may be related to poor prognosis in such patients. CAR, however, is a more valuable parameter than NLR in terms of indicating poor prognosis
Kim et al. (2020) [18]	Retrospective	84 patients	47 ± 14.35 (Mean)	ELR, NLR, BLR	A higher pretreatment NLR and neutrophil count and a lower day-7 lymphocyte count were observed in patients with RHS with unfavorable outcomes. In patients with BP, both the initial HB grade and the pretreatment eosinophil count were included
Baklacı et al. (2020) [19]	Retrospective	76 patients, 60 controls	39.76 ± 9.02, 39.31 ± 8.16 (Mean)	NLR, PLR	Mean LDH concentrations and NLR were significantly high in BP group than in control group
Cayir et al. (2020) [20]	Retrospective, archival, cross-sectional study	79 patients (23 non recovery group patients, 56 recovery group patients), 60 controls	49 ± 3 (Non recovery), 49 ± 3 (Recovery), 50 ± 3 (Controls)	WBC, CRP, neutrophil, lymphocyte, NLR, CAR, PLR	Higher CAR and NLR were associated with poor prognosis in BP. CAR might be the most significant indicator of poor prognosis in BP
Kınar et al. (2020) [21]	Retrospective	88 patients,50 controls	54,47 (Mean)	Neutrophil, lymphocytes, NLR, PLR	Patient group had higher neutrophil counts and higher SII and NLR values than the control group. Among BP patients, the SII values of the unrecovered group were statistically higher than those of the recovered group
Aysel et al. (2020) [22]	Retrospective	47 patients	14.7 ± 2.5	NLR, TLR	NLR in patients with advanced grades (grade 4, 5, 6) was higher, compared to that of patients with grade 2 and 3. NLR at admission can be considered as a prognostic factor
Demir et al. (2020) [23]	Prospective	92 patients, 90 Controls	38 ± 9.5, 41.7 ± 17.2 (Mean)	Cyclophilin A	Low CyPA indicates the shorter average recovery time than that of high CyPA
Kim et al. (2020) [24]	Retrospective	54 patients, 39 controls	14.5 ± 4.1, 11.9 ± 5.2 (Mean)	NLR, PLR	The NLR and PLR values in the BP group were significantly higher than in the control group. However, there were no significant differences between the low-grade and high-grade BP groups nor were there any statically significant differences in the other characteristics.
Kim et al. (2019) [25]	Retrospective	51 patients	39.7 ± 20.1 (Mean)	WBC, NLR, PLR	NLR was significantly higher in patients with severe than with mild to moderate palsy. Recovery time was significantly longer in patients with high NLR than low NLR
Soh et al. (2019) [26]	Retrospective	102 patients	45.1 ± 16	NLR, PLR	The HBS grade of the high-NLR group was significantly higher than that of the normal-NLR group. Patients with RHS who have an elevated NLR have poor outcomes in terms of the HBS grade
Karatoprak et al. (2019) [27]	Retrospective	102 patients	10.37 ± 4.2 (Mean)	NLR	NLR and RDW were not found to be predictive factors for early recovery
Ulusoy et al. (2018) [28]	Prospective	24 patients, 29 controls	45.12 ± 12.34, 44.34 ± 9.97 (Mean)	NLR, PLR	There was no correlation between the NLR value and the prognosis of Bell’s Palsy. A comparison of the recovered and unrecovered patients revealed that the PDW value was significantly higher and the platelet count was lower in the recovered patients than the unrecovered patients
Horibe et al. (2017) [29]	Retrospective	61 patients	16-50 years	NLR	RDW can predict recovery from BP in patients aged 50 years and less
Wasano et al. (2017) [7]	Retrospective	468 patients (BP 374, RHS 94)	BP Mean 51.7 ± 14.18.8 (recovered), 57.6 ± 16.6 (unrecovered) RHS Mean 50.8 ± 18.7 (recovered), 50.5 ± 18.0 (unrecovered)	NLR, Monocyte rate	Palsy Prognosis Prediction scores (PPP score) are useful for predicting prognosis of facial palsy before beginning treatment
Sahin et al. (2017) [30]	Retrospective	28 patients, 28 controls	29.5 ± 10.5 (Mean)	NLR, PLR	Significant changes were not observed in NLR, PLR, MPV and RDW measurements in BP group between HBS
Kilicaslan et al. (2016) [31]	Prospective	54 patients (32 low-grade group & 22 high-grade group), 35 controls	Mean BP 39.1 ± 14.2 (Low-grade), 36.9 ± 15.9 (high-grade), 38.6 ± 18.1 (Controls)	Procalcitonin	Procalcitonin levels were significantly associated with the severity of BP and higher PCT levels were found to be related with poor prognosis
Wasano et al. (2016) [32]	Retrospective	468 patients (BP 374, RHS 94)	Mean BP 51.7 ± 14.18.8 (recovered), 57.6 ± 16.6 (unrecovered) Mean RHS 50.8 ± 18.7 (recovered), 50.5 ± 18.0 (unrecovered)	NLR, WBC	In the BP group, neutrophil rate, lymphocyte rate, NLR of recovered patients were significantly different than those of unrecovered patients. In RHS group monocyte rate, platelet count, MCV of recovered patients were significantly different than those of unrecovered patients
Kiliçkaya, et al. (2015) [33]	Retrospective	146 patients, 140 controls	HBS grade I–II (38.9 ± 22.77), HBS grade III–IV (36.04 ± 21.77), HBS grade V-VI (42.30 ± 17.43) (Mean)	NLR	As the severity of the paralysis increased in the APFP patients in this study, the NLR value increased. the NLR value can be used as an early predictive prognostic factor of IPFP
Eryilmaz et al. (2015) [34]	Retrospective	25 patients, 25 controls	9.86 ± 5.07, 9.14 ± 5.94 (Mean)	NLR	NLR and pretreatment HBS showed no statistically significant correlation
Atan et al. (2015) [35]	Retrospective	99 patients, 99 controls	47.84 ± 16.94, 44.22 ± 8.64 (Mean)	NLR, PLR	No statistically significant relation was detected between the degree of facial paralysis and NLR and PLR
Kum et al. (2014) [36]	Retrospective	65 patients, 35 controls	45 ± 3.2, 45.4 ± 4.1 (Mean)	NLR	There was a positive and significant correlation between NLR and HBS of the patients. MPV did not show any significant correlation with any of the parameters studied
Özler, et al. (2014) [37]	Prospective	25 patients, 25 controls	40.7 ± 12.3, 39.7 ± 8.26 (Mean)	NLR	A positive correlation between NLR values and grade of facial paralysis
Bucak et al. (2013) [38]	Retrospective	54 patients, 45 controls	43.11 ± 18.12, 48.33 ± 5.65 (Mean)	NLR	The mean NLR levels were higher in unsatisfactory recovered patients compared with satisfactory recovered ones

**Table 2:** Summary of prognostic biomarkers for acute peripheral facial palsy (Metabolic parameter).

Author (Year)	Study Design	Sample Size	Age (years)	Biomarkers	Results/Conclusions
Baklacı et al. (2020) [19]	Retrospective	76 patients, 60 controls	39.76 ± 9.02, 39.31 ± 8.16 (Mean)	LDH	Mean LDH concentrations and NLR were significantly high in BP group than in control group
KARAGÖZ et al. (2020) [39]	Prospective	86 patients, 28 controls	41, 38 (Mean)	IR, HOMA-IR	The patients with higher HOMA-IR values had more severe facial dysfunction at the initial presentation and complete recovery time took longer than the patients with normal HOMA-IR value. Recovery time was significantly longer in prediabetics and newly diagnosed diabetic patients than in patients with normal glycemia
Jung et al. (2018) [40]	Retrospective	124 patients	52.16 ± 14.17 (Mean)	TG, HDL-C	The recovery rate of BP was significantly lower in the MetS group than in the Non-MetS group, particularly affected by high TG
Wasano et al. (2016) [32]	Retrospective	468 patients (BP 374, RHS 94)	Mean BP 51.7 ± 14.18.8 (recovered), 57.6 ± 16.6 (unrecovered) Mean RHS 50.8 ± 18.7 (recovered), 50.5 ± 18.0 (unrecovered)	HbA1c	In the BP group, neutrophil rate, lymphocyte rate, NLR of recovered patients were significantly different than those of unrecovered patients. In RHS group monocyte rate, platelet count, MCV of recovered patients were significantly different than those of unrecovered patients
Riga et al. (2012) [41]	Prospective	56 patients	54 ± 31.7 (Mean)	HbA1c	The 20 patients with abnormal HbA1c values were more frequently diagnosed with BP of grade V/VI. However, their HBS were not found to be worse at the 6-month follow-up visit
Kanazawa et al. (2007) [42]	Prospective	76 patients	64.6 ± 8.5 (Diabetic group), 61.3 ± 8.5 (Nondiabetic group) (Mean)	HbA1c	Facial movement in the DG was poorer than that in the NDG at 3 months and 6 months after onset. In terms of the recovery rate, the rate in the DG was much lower than that in the NDG at 6 months after onset

**Table 3:** Summary of prognostic biomarkers for acute peripheral facial palsy (Hemostatic parameter).

Author (Year)	Study Design	Sample Size	Age (years)	Biomarkers	Results/Conclusions
Cayir et al. (2021) [17]	Retrospective	59 patients, 65 controls	10.69 ± 5.76, 10.87 ± 3.22 (Mean)	MPV, Hbg, RDW	High NLR and CAR values in pediatric BP may be related to poor prognosis in such patients. CAR, however, is a more valuable parameter than NLR in terms of indicating poor prognosis
Baklacı et al. (2020) [19]	Retrospective	76 patients, 60 controls	39.76 ± 9.02, 39.31 ± 8.16 (Mean)	MPV	Mean LDH concentrations and NLR were significantly high in BP group than in control group
Cayir et al. (2020) [20]	Retrospective, archival, cross-sectional study	79 patients (23 non recovery group patients, 56 recovery group patients), 60 controls	49 ± 3 (Non recovery), 49 ± 3 (Recovery), 50 ± 3 (Controls)	Platelet, Hbg	Higher CAR and NLR were associated with poor prognosis in BP. CAR might be the most significant indicator of poor prognosis in BP
Kınar et al. (2020) [21]	Retrospective	88 patients, 50 controls	54, 47 (Mean)	Platelet	Patient group had higher neutrophil counts and higher SII and NLR values than the control group. Among BP patients, the SII values of the unrecovered group were statistically higher than those of the recovered group
Aysel et al. (2020) [22]	Retrospective	47 patients	14.7 ± 2.5	MPV	NLR in patients with advanced grades (grade 4, 5, 6) was higher, compared to that of patients with grade 2 and 3. NLR at admission can be considered as a prognostic factor
Kim et al. (2020) [24]	Retrospective	54 patients, 39 controls	14.5 ± 4.1, 11.9 ± 5.2 (Mean)	MPV, RDW	The NLR and PLR values in the BP group were significantly higher than in the control group. However, there were no significant differences between the low-grade and high-grade BP groups nor were there any statically significant differences in the other characteristics.
Kim et al. (2019) [25]	Retrospective	51 patients	39.7 ± 20.1 (Mean)	Platelet	NLR was significantly higher in patients with severe than with mild to moderate palsy. Recovery time was significantly longer in patients with high NLR than low NLR
Ulusoy et al. (2018) [28]	Prospective	24 patients, 29 controls	45.12 ± 12.34, 44.34 ± 9.97 (Mean)	PDW, platelet	There was no correlation between the NLR value and the prognosis of Bell’s Palsy. A comparison of the recovered and unrecovered patients revealed that the PDW value was significantly higher and the platelet count was lower in the recovered patients than the unrecovered patients
Horibe et al. (2017) [29]	Retrospective	61 patients	16–50 years	MPV, RDW	RDW can predict recovery from BP in patients aged 50 years and less
Wasano et al. (2017) [7]	Retrospective	468 patients (BP 374, RHS 94)	BP Mean 51.7 ± 14.18.8 (recovered), 57.6 ± 16.6 (unrecovered) RHS Mean 50.8 ± 18.7 (recovered), 50.5 ± 18.0 (unrecovered)	Platelet, MCV	Palsy Prognosis Prediction scores (PPP score) are useful for predicting prognosis of facial palsy before beginning treatment
Sahin et al. (2017) [30]	Retrospective	28 patients, 28 controls	29.5 ± 10.5 (Mean)	MPV, RDW	Significant changes were not observed in NLR, PLR, MPV and RDW measurements in BP group between HBS
Zhao et al. (2016) [43]	Retrospective	105 patients, 22 controls		Fibrinogen	The plasma fibrinogen concentration was significantly higher in the group of patients with BP (HBS IV-VI) than the in the control group. The plasma fibrinogen levels became higher with the HBS grading increase
Wasano et al. (2016) [32]	Retrospective	468 patients (BP 374, RHS 94)	Mean BP 51.7 ± 14.18.8 (recovered), 57.6 ± 16.6 (unrecovered) Mean RHS 50.8 ± 18.7 (recovered), 50.5 ± 18.0 (unrecovered)	Platelet, Hbg, MCV	In the BP group, neutrophil rate, lymphocyte rate, NLR of recovered patients were significantly different than those of unrecovered patients. In RHS group monocyte rate, platelet count, MCV of recovered patients were significantly different than those of unrecovered patients
Özler, et al. (2014) [14]	Prospective	30 patients, 30 controls	39.9 ± 10.68, 37.1 ± 6.91 (Mean)	MPV, PDW, platelet	There was positive correlation between MPV values and grade of facial paralysis. Also, there was positive correlation between PDW values and grade of facial paralysis. In contrast, there was no correlation between MPV and PDW values and prognosis of facial paralysis
Kum et al. (2014) [36]	Retrospective	65 patients, 35 controls	45 ± 3.2, 45.4 ± 4.1 (Mean)	MPV	There was a positive and significant correlation between NLR and HBS of the patients. MPV did not show any significant correlation with any of the parameters studied

**Table 4:** Summary of prognostic biomarkers for acute peripheral facial palsy (Immunologic parameter).

Author (Year)	Study Design	Sample Size	Age (years)	Biomarkers	Results/Conclusions
Kaygusuz et al. (2004) [44]	Prospective	30 patients, 30 controls	38.7 ± 15.3 (Mean)	TNF-α, IFN-γ, TGF-β, CD3+, CD4+, CD8+, CD19+, CD16+ plus 56+	CD4+ cell and ratio of CD4+/CD8+ lower and the percentage of the CD8+ and CD16+ plus 56+ cells higher compared with the control group. The levels of TNF-α were lower, whereas IFN-γ and TGF-β1 were higher
Tekgul et al. (2004) [15]	Prospective	17 patients, 20 controls	7.82 ± 4.41, 12.4 ± 8.4	Immunologic parameters (total T cells (CD3+), B cells (CD19+), T helper/inducer cells (CD4+), T cytotoxic (CD8+), and natural killer cells (CD16+ CD56+)	We did not find any prognostic significance of lymphocyte subset analysis in peripheral blood to predict outcome in patients with unsatisfactory recovery
Yılmaz et al. (2002) [45]	Prospective	23 patients, 30 controls	40.2 ± 15.7, 42.4 ± 8.4 (Mean)	Immunologic parameters (IL-1β, IL-2r, IL-6, IL-8, and TNF-α)	The cytokine levels of did not correlate with the degree of recovery

**Table 5:** Summary of prognostic biomarkers for acute peripheral facial palsy (Oxidative parameter).

Author (Year)	Study Design	Sample Size	Age (years)	Biomarkers	Results/Conclusions
Çalcı et al. (2018) [46]	Prospective	30 patients, 30 controls	33.6 ± 8.3, 31.1 ± 6.4 (Mean)	PON, ARE, IMA, albumin-adjusted IMA	PON and ARE levels of the patient group were significantly lower than controls and IMA, albumin-adjusted IMA levels were significantly higher than controls
Babademez et al. (2016) [12]	Prospective	77 patients, 38 controls	38.48 ± 10.31, 37.37 ± 10.75 (Mean)	Native thiol (-SH) and total thiol (-SH+-S-S-)	The mean native thiol and total thiol were significantly lower and disulfide levels were higher in BP than controls. However, there was no correlation between the HBS and thiol profiles

## Data Availability

Not applicable.

## Abbreviations

NLR	Neutrophil to lymphocyte ratio
PLR	Platelet to lymphocyte ratio
CAR	C-reactive protein to albumin ratio
MPV	Mean platelet volume
RDW	Red cell distribution width
Hbg	Hemoglobin
ELR	Eosinophil to lymphocyte ratio
BLR	Basophil to lymphocyte ratio
LDH	Lactate dehydrogenase
WBC	White blood cell
TLR	Thrombocyte to lymphocyte ratio
SII	Systemic Immune-Inflammation Index, SII = platelets × neutrophils/lymphocytes
CyPA	Cyclophilin A
IR	Insulin resistance
HOMA	Homeostasis model assessment
HOMA-IR	basal plasma glucose (mg/dL) × basal plasma insulin (UI/mL)/405
TG	Triglyceridemia
HDL-C	High density lipoprotein cholesterol
PON	Paraoxonase
ARE	Arylesterase
IMA	Ischemia modified albumin
MCV	Mean corpuscular volume
HBS	House-Brackmann facial nerve grading system
BP	Bell’s palsy
RHS	Ramsay-Hunt syndrome
IPFP	Idiopathic peripheral facial palsy
PDW	Platelet distribution width
HbA1c	Serum glycosylated hemoglobin
DG	Diabetic group
NDG	Non diabetic group
IL	Interleukin
TNF-α	Tumor necrosis factor alpha
